# Severe Obesity Shifts Metabolic Thresholds but Does Not Attenuate Aerobic Training Adaptations in Zucker Rats

**DOI:** 10.3389/fphys.2016.00122

**Published:** 2016-04-19

**Authors:** Thiago S. Rosa, Herbert G. Simões, Marcelo M. Rogero, Milton R. Moraes, Benedito S. Denadai, Ricardo M. Arida, Marília S. Andrade, Bruno M. Silva

**Affiliations:** ^1^Graduate Program in Translational Medicine, Federal University of São PauloSão Paulo, Brazil; ^2^Graduate Program in Physical Education and Health, Catholic University of BrasíliaBrasília, Brazil; ^3^Department of Nutrition, School of Public Health, University of São PauloSão Paulo, Brazil; ^4^Department of Nephrology, Federal University of São PauloSão Paulo, Brazil; ^5^Human Performance Laboratory, Department of Physical Education, São Paulo State UniversityRio Claro, Brazil; ^6^Department of Physiology, Federal University of São PauloSão Paulo, Brazil

**Keywords:** aerobic capacity, exercise training, diabetes, obesity, maximal lactate steady state, incremental test

## Abstract

Severe obesity affects metabolism with potential to influence the lactate and glycemic response to different exercise intensities in untrained and trained rats. Here we evaluated metabolic thresholds and maximal aerobic capacity in rats with severe obesity and lean counterparts at pre- and post-training. Zucker rats (obese: *n* = 10, lean: *n* = 10) were submitted to constant treadmill bouts, to determine the maximal lactate steady state, and an incremental treadmill test, to determine the lactate threshold, glycemic threshold and maximal velocity at pre and post 8 weeks of treadmill training. Velocities of the lactate threshold and glycemic threshold agreed with the maximal lactate steady state velocity on most comparisons. The maximal lactate steady state velocity occurred at higher percentage of the maximal velocity in Zucker rats at pre-training than the percentage commonly reported and used for training prescription for other rat strains (i.e., 60%) (obese = 78 ± 9% and lean = 68 ± 5%, *P* < 0.05 vs. 60%). The maximal lactate steady state velocity and maximal velocity were lower in the obese group at pre-training (*P* < 0.05 vs. lean), increased in both groups at post-training (*P* < 0.05 vs. pre), but were still lower in the obese group at post-training (*P* < 0.05 vs. lean). Training-induced increase in maximal lactate steady state, lactate threshold and glycemic threshold velocities was similar between groups (*P* > 0.05), whereas increase in maximal velocity was greater in the obese group (*P* < 0.05 vs. lean). In conclusion, lactate threshold, glycemic threshold and maximal lactate steady state occurred at similar exercise intensity in Zucker rats at pre- and post-training. Severe obesity shifted metabolic thresholds to higher exercise intensity at pre-training, but did not attenuate submaximal and maximal aerobic training adaptations.

## Introduction

In humans, the lactate (LT) and glycemic (GT) thresholds are usually identified at similar exercise intensity, which precedes the intensity where the maximal lactate steady state (MLSS) occurs (Simões et al., 1999, 2003; Billat et al., 2003; Beneke et al., 2011). The LT and MLSS have been largely used to distinguish boundaries of metabolic domains, such that exercise intensity is considered moderate below the LT, heavy between the LT and the MLSS, and severe above the MLSS (Beneke et al., 2011). Nonetheless, these human boundaries do not seem to be applicable to rodents, which is an issue that has received little attention. So far, there is evidence that the exercise intensity where blood lactate accumulation begins (i.e., LT), determined by visual inspection or polynomial adjustment, coincides with the MLSS intensity in healthy rats and ob/ob mice (Voltarelli et al., 2002; Cunha et al., 2009; Almeida et al., 2011). However, it is currently unknown whether rats with severe obesity (e.g., obese Zucker rats) also show the MLSS, LT, and GT at similar exercise intensity. In addition, remains to be investigated whether the LT and GT identification is feasible and what is the best method for its identification in Zucker rats.

Most of the studies that investigated the effect of aerobic exercise training in obese Zucker rats did not assess aerobic capacity by an exercise test (Frisbee et al., 2006; Peterson et al., 2008; Barretti et al., 2012; Garcia et al., 2013; Martin-Cordero et al., 2013; You et al., 2013; Disanzo and You, 2014). Therefore, exercise training was conducted without precise data to determine exercise intensity according to metabolic domains. For some rat and mice strains (e.g., Wistar, Diabetic rats and C57/6J mice), maximal velocity (Vmax) achieved on an incremental treadmill test has been used to estimate the maximal oxygen consumption and to determine exercise intensity for training protocols (De Angelis et al., 2004; Irigoyen et al., 2005; Rodrigues et al., 2007). In these cases, 60% of the Vmax approximately represents the intensity of the MLSS (Rodrigues et al., 2007; Teixeira et al., 2012). Nevertheless, such intensity is probably not adequate to prescribe exercise for Zucker rats. The reason for that is that obese Zucker rats have many characteristics that resemble humans with morbid obesity (Aleixandre de Artiñano and Miguel Castro, 2009), and these subjects show ventilatory threshold at exercise intensity higher than 60% of maximal aerobic capacity (Li et al., 2001). Thus, obese Zucker rats may follow this pattern.

The effect of aerobic training on submaximal and maximal aerobic capacity in Zucker rats is also unknown. Only one study has assessed the effect of aerobic training on the MLSS in obese Zucker rats (Almeida et al., 2014), and reported these rats showed attenuated training-induced increase in submaximal aerobic capacity. Nevertheless, this study has a key limitation, since Zucker rats were compared to Wistar Kyoto rats. These rat strains have different genetic background, and thus it is not possible to interpret whether the attenuated training-induced increase in submaximal aerobic capacity was attributed to dissimilar genetic background or to obesity *per se*. Given aerobic training-induced increase in citrate synthase activity is similar between lean and obese Zucker rats (You et al., 2013), it is plausible that the effect of aerobic training on submaximal and maximal aerobic capacity is not attenuated in obese Zucker rats, as previously suggested (Almeida et al., 2014).

Based on this background, the aims of this study were to (1) compare the exercise intensity of the MLSS with the exercise intensity of the LT and GT at pre- and post-training; (2) identify the percentage that the MLSS occurs in relation to the Vmax at pre- and post-training; and (3) investigate the effect of aerobic training on the MLSS, LT, GT and Vmax in obese and lean Zucker rats. We hypothesized that (1) the MLSS, LT and GT would occur at comparable exercise intensity at pre- and post-training; (2) severe obesity would shift the MLSS to higher percentage of the maximal aerobic capacity at pre- and post-training; and (3) the effect of aerobic training on MLSS, LT, GT and Vmax would be similar between obese and lean rats.

## Methods

### Animals

Ten male obese Zucker rats (*fa*^−^*/fa*^−^) and 10 lean counterparts (*Fa*^+^*/fa*^−^) were purchased from the Federal University of São Paulo (CEDEME/UNIFESP). Rats were 12 weeks old, housed in collective cages (2–4 animals/cage) at temperature of 22 ± 2°C and relative humidity of 55 ± 10%, light/dark cycle of 12 h/12 h (lights on at 06:00 a.m.), with food (Nuvital CR1, Nuvilab, Brazil) and water *ad libitum*. This study was approved by the Ethics Committee for animal use of the Federal University of São Paulo/UNIFESP (No. 1852/11). All procedures were in accordance with the Brazilian College of Animal Experimentation (COBEA).

### Experimental design

The study began with treadmill running familiarization, according with protocol adapted from Copp et al. (2009). Rats ran at 6 m/min, 10 min per day, 5 days a week, during 2 weeks on a treadmill with individual lanes and electrical stimulation at the rear (customized model, AVS Projects, Brazil). Next, rats underwent constant load tests for MLSS identification and thereafter an incremental test for LT, GT, and Vmax identification (Simões et al., 1999, 2003; Cunha et al., 2009; Almeida et al., 2011, 2013, 2014; Petriz et al., 2013). Then, rats underwent aerobic training, and in the end, exercise tests were repeated. Familiarization, tests and training were performed during the dark phase of the 24 h cycle (exercise sessions started at 6:30 p.m.). Blood samples and exercise tests were done after 4 h of fasting.

### Maximal lactate steady state

Three constant velocity tests were performed on different days, with a minimum interval of 48 h to determine the MLSS velocity. Workloads ranged from 10 m/min to 14 m/min for obese rats and from 14 m/min to 18 m/min for lean rats at pre-training. These ranges were defined with basis on a pilot protocol, in which rats were submitted to 4–5 different workloads. Results from this pilot protocol showed that three workloads were enough to determine the MLSS velocity, similarly to previous studies (Almeida et al., 2013, 2014). Treadmill grade was set at 0%. Velocity of the first test was randomly chosen. Then, velocity of the next two tests was set according with the lactate response of the previous test (e.g., if lactate did not rise progressively in the first test, velocity of the second test was higher than the first one). Blood samples were drawn at 0 or rest, 10, 20, and 30 min. At post-training, the first test was done at the highest velocity used in pre-training tests, and range was changed (14–18 m/min for obese rats and 18–22 m/min for lean rats). The criterion used to identify the MLSS velocity was the highest velocity that yielded no change or increase of blood lactate up to 0.5 mM during the last 10 min of exercise, according with the methods reported by Aunola and Rusko (1992).

### Lactate and glycemic thresholds

The incremental test was conducted at 0% grade. The test started at 6 m/min and velocity increased 2 m/min every 3 min until exhaustion (i.e., when the animal could not maintain running velocity, stopping consecutive times; Copp et al., 2009). One-minute interval was used between each increment in velocity for blood collection. This protocol was adapted from Gobatto et al. (2001) and Almeida et al. (2011). The LT and GT velocities were identified as follows: (1) visual inspection (LTv and GTv) by two independent assessors, with previous experience to identify the inflexion in the curve of [Lac] and [Gluc] plotted vs. velocity (Beaver et al., 1985; Cheng et al., 1992), who were blinded to the MLSS data. Identification of these assessors coincided in most of the cases, but when identification was dissimilar, the assessors reviewed the curves together to get an agreement. Afterward a third blinded assessor also identified the LTv and GTv, which was compared to the identification of the other assessors; (2) polynomial adjustment of the ratio between [Lac] or [Gluc] and velocity (Qlac or Qgluc) plotted against the velocity of each incremental stage. Then, a second order polynomial function was used, which derivate allowed identification of the velocity corresponding to the lowest point in the curve of Qlac or Qgluc vs. velocity. These points were considered the LTp (Almeida et al., 2011) and GTp (Simões et al., 1999, 2003), respectively; (3) mathematical models for the LT assessment through the Software Lactate E 2.0 (LTs, Log-log LTs, and Dmax) (Beaver et al., 1985; Lundberg et al., 1986; Cheng et al., 1992; Newell et al., 2007). The value of the LTs was identified at the intersection of two linear regressions on a plot of [Lac] vs. velocity (Lundberg et al., 1986). These linear regressions were defined automatically considering the combination of data that resulted in the least amount of residuals. The Log-log LTs consisted on the logarithmic transformation (Log_10_) of the [Lac] and velocity, and then the LTs was calculated (Beaver et al., 1985). Dmax was the velocity corresponding to the farthest perpendicular point from a line joining the first and last lactate measurements, which was estimated using a linear regression (Cheng et al., 1992). Vmax was obtained in the incremental test, as the velocity of the last complete stage supported by the animal.

### Blood collection and analysis

After local antisepsis with 70% alcohol, 25 μL of blood were collected from a small incision in the distal tail portion using a calibrated capillary tube. The blood sample was rapidly deposited in Eppendorf® microtubes (0.6 mL), containing 50 μL of 1% sodium fluoride (NaF), and stored at −80°C for further biochemical analysis. The electroenzymatic method (YSI 2300, Yellow Springs Instruments, USA) was utilized to determine [Lac] and [Gluc] (Cunha et al., 2009; Almeida et al., 2011).

### Aerobic training

Rats ran continuously at the MLSS velocity, with 0% grade, 5 days per week, for 8 weeks. Exercise sessions lasted 20 min in the first day of the training period and duration increased 10 min each 2 days. At the end of the 10th day animals ran continuously for 60 min, until the end of the 8th week. Exercise tests were repeated 2 days after the last training session.

### Statistical analyses

Data are presented as mean ± SD. Groups were compared at pre- and post-training by two-way ANOVA, followed by the Fisher's *post-hoc*. The MLSS velocity, as the percentage of the Vmax, was compared to the fixed value of 60% by the one sample Student's *t*-test. The MLSS velocity was compared with each of the other methods (LTv, LTp, GTv, GTp, LTs, Log-log LTs, and Dmax) via the paired Student's *t*-test. The response to aerobic training [(post – pre)/pre × 100)] was also compared via the paired Student's *t*-test. Association between the MLSS velocity with other methods was analyzed by the Bland-Altman plot (Bland and Altman, 1986) and intraclass correlation coefficient (ICC). Statistical significance was accepted at *P* < 0.05. Statistical analyses were performed using the SPSS 19.0 software (SPSS Inc., USA).

## Results

Body weight of the obese group was higher than the lean group at pre-training (Table 1). At post-training, body weight did not change in the lean group (*P* > 0.05), but increased approximately 5% in the obese group (*P* < 0.05). Resting [Lac] of the obese group was higher than the lean group, but there was no difference in resting [Lac] within groups at pre- vs. post-training (Table 1). Resting [Glu] was similar between groups at pre-training and decreased in both groups at post-training (Table 1).

**Table 1:** **Body weight, resting glucose and resting lactate at pre- and post-training**.

	**Lean group**		**Obese group**		**ANOVA main effects**		
	**Pre-training**	**Post-training**	**Pre-training**	**Post-training**	**Group**	**Time**	**Interaction**
Body weight (g)	387.9 ± 6.7	379.6 ± 13.4	537.5 ± 50.1^a^^,^^b^	564.5 ± 45.9^a^^,^^b^^,^^c^	< 0.01	0.07	< 0.01
Resting glucose (mg/dL)	114.4 ± 8.82	102.7 ± 7.93	124.6 ± 7.77	105.6 ± 10.97	0.05	< 0.01	0.15
Resting lactate (mM)	3.08 ± 0.88	2.74 ± 0.77	3.92 ± 0.55	3.88 ± 0.71	< 0.01	0.33	0.44

a P < 0.05 vs. lean pre;

b P < 0.05 vs. lean post; and

c *P < 0.05 vs. obese pre*.

**Figure 1:**
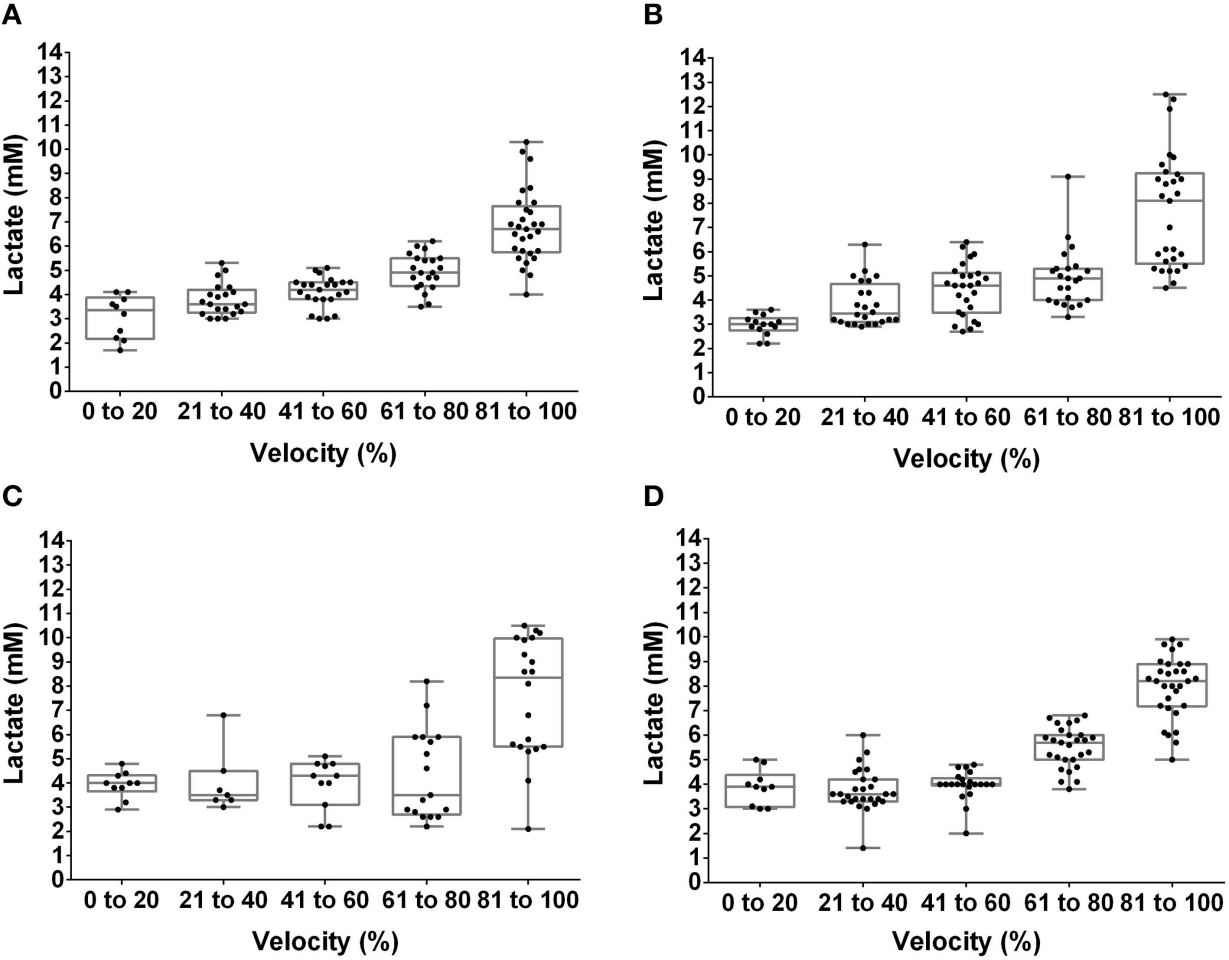
**Box plot and dispersion of the lactate concentration during the incremental test in lean rats at pre- (A) and post-training (B) and obese rats at pre- (C) and post-training (D)**.

**Figure 2:**
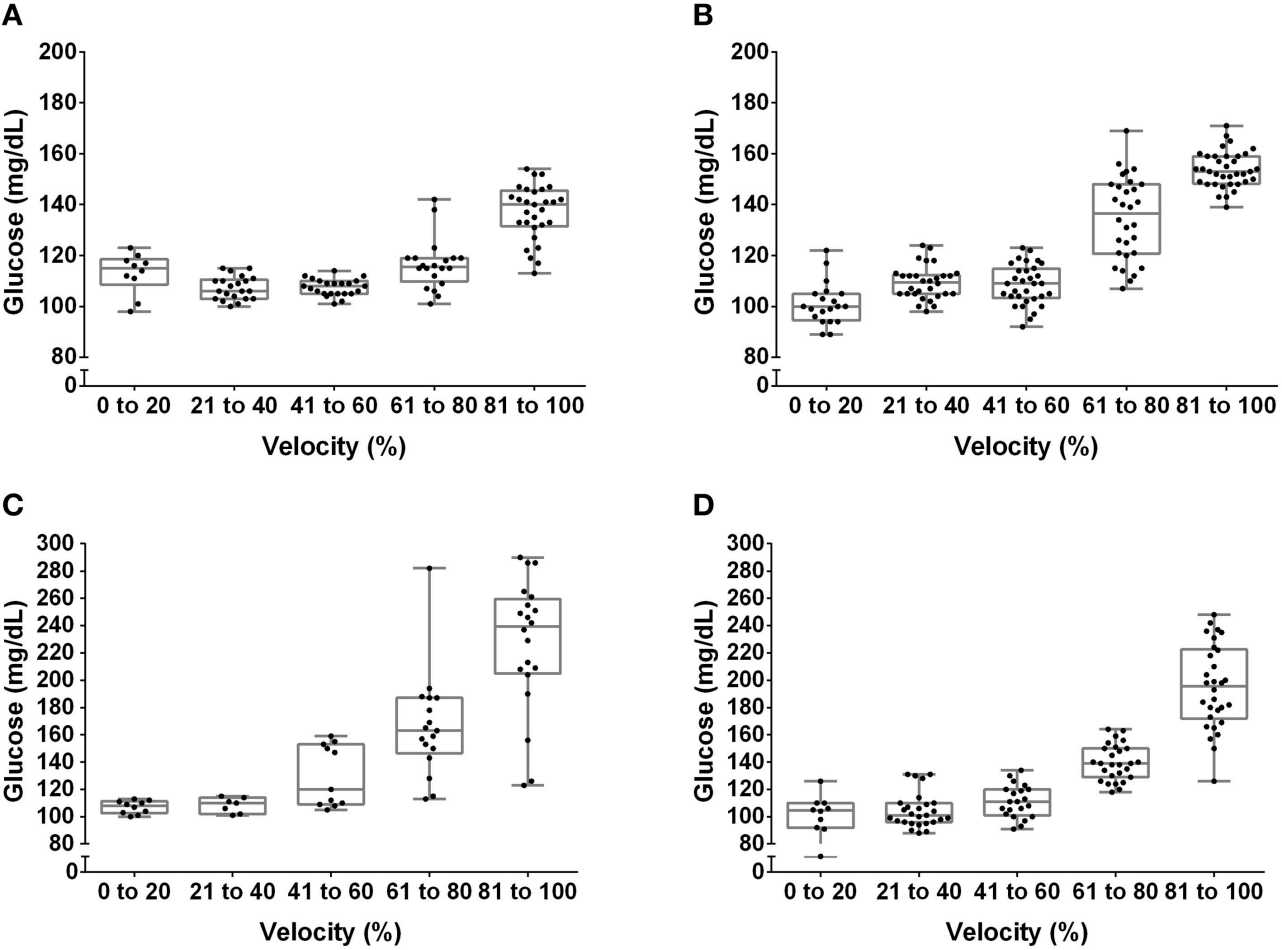
**Box plot and dispersion of the glucose concentration during the incremental test in lean rats at pre- (A) and post-training (B) and obese rats at pre- (C) and post-training (D)**.

**Figure 3:**
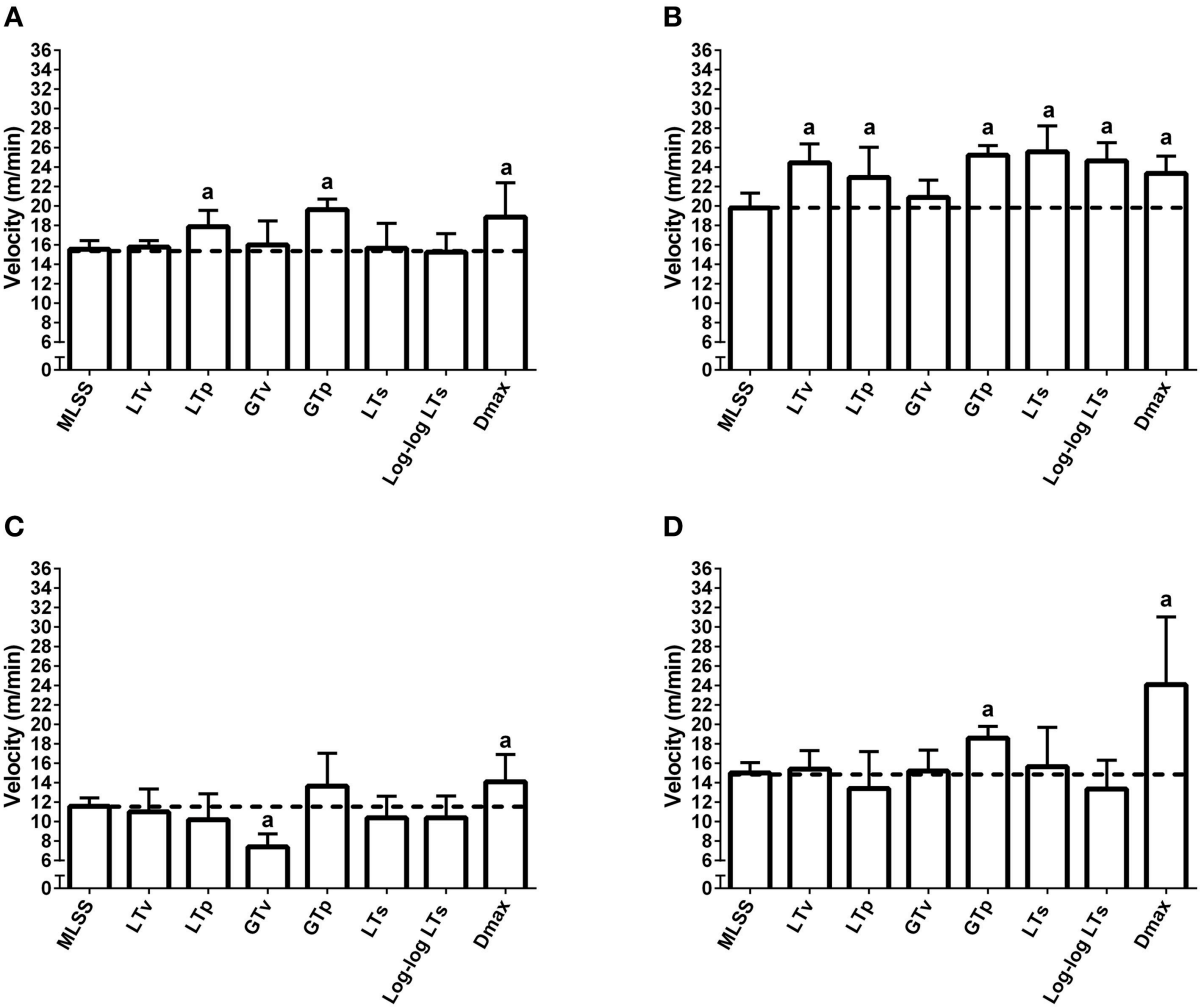
**Maximal lactate steady state (MLSS), lactate threshold (LT) and glycemic threshold (GT) velocities in lean rats at pre- (A) and post-training (B) and obese rats at pre- (C) and post-training (D)**. ^*a*^*P* < 0.05 vs. the MLSS velocity within each group. The dashed lines mark the mean of the MLSS.

The [Lac] at the MLSS was 3.38 ± 0.27 mM, 3.18 ± 0.51 mM, 3.69 ± 0.37 mM, and 3.82 ± 0.36 mM, for lean pre-, lean post-, obese pre- and obese post-training, respectively (*P* > 0.05). Figures 1, 2 show the [Lac] and [Gluc] data, respectively, from all rats during the incremental protocol. From the box plots it is clear that data dispersion increases toward the end of the protocol. Figure 3 shows comparison between the MLSS velocity and the LT and GT velocities in both groups, at pre- and post-training. The LTv, GTv, LTs, and Log-log LTs velocities corresponded to the MLSS velocity in three within group comparisons (*P* > 0.05 vs. MLSS). The LTp velocity corresponded to the MLSS velocity in two within group comparisons (*P* > 0.05 vs. MLSS). The GTp velocity corresponded to the MLSS velocity in only one within group comparison (*P* > 0.05 vs. MLSS). Lastly, the Dmax velocity did not correspond to the MLSS velocity in any comparison (*P* < 0.05 vs. MLSS). Noteworthy, when the LT or GT velocities did not coincide with the MLSS velocity, the velocity of the LT and GT was higher than the MLSS velocity in all cases (*P* < 0.05), except one (GTv obese pre).

**Figure 4:**
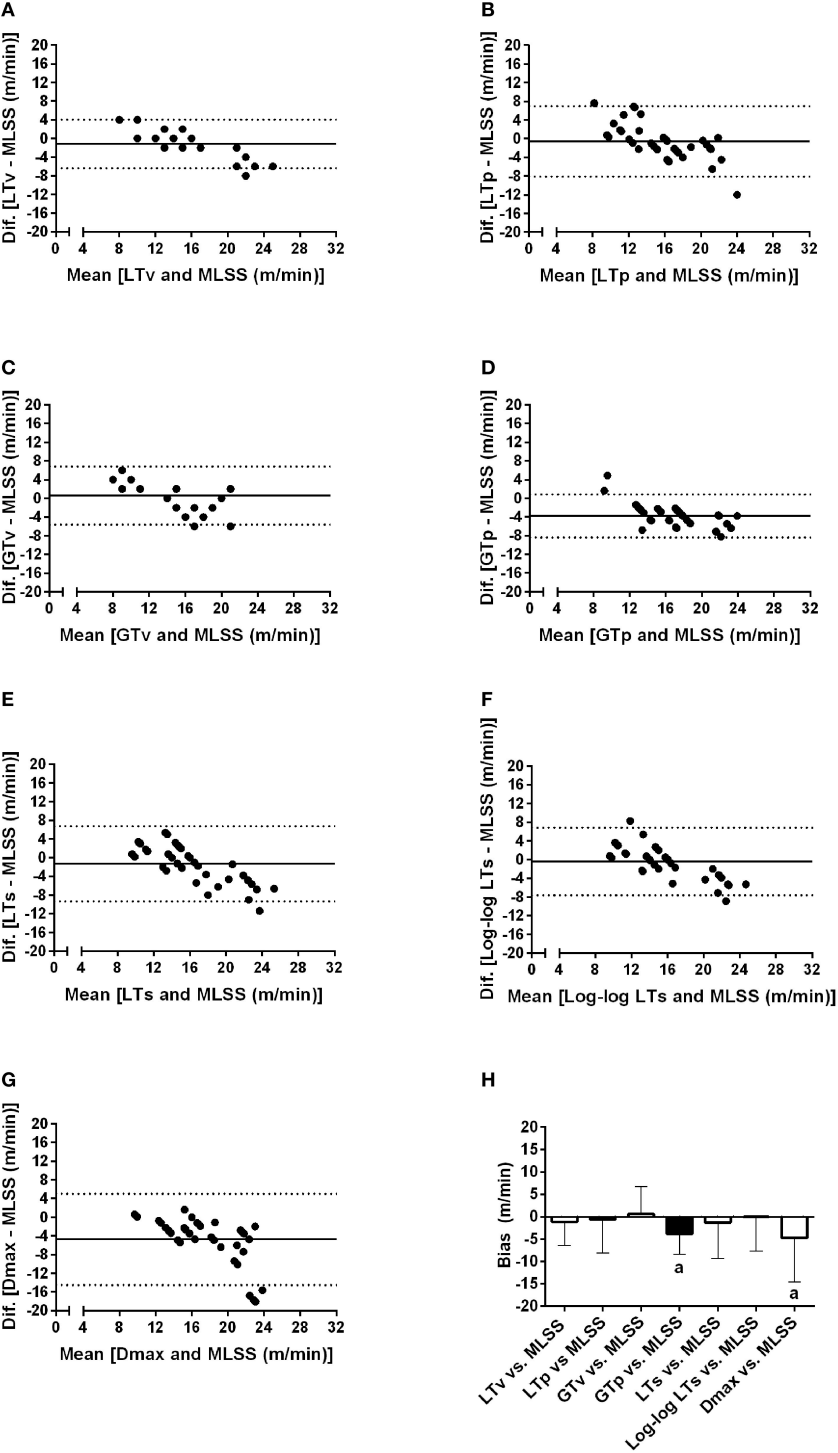
**Bland-Altman plots of the lactate threshold (LT) and glycemic threshold (GT) velocities vs. the maximal lactate steady state (MLSS) velocity**. **(A–G)** Individual analysis of the concordance between each method and maximal lactate steady state velocity through the Bland and Altman plots. **(H)** shows the average and standard deviation of the bias for each method. ^*a*^*P* < 0.05 vs. zero.

**Figure 5:**
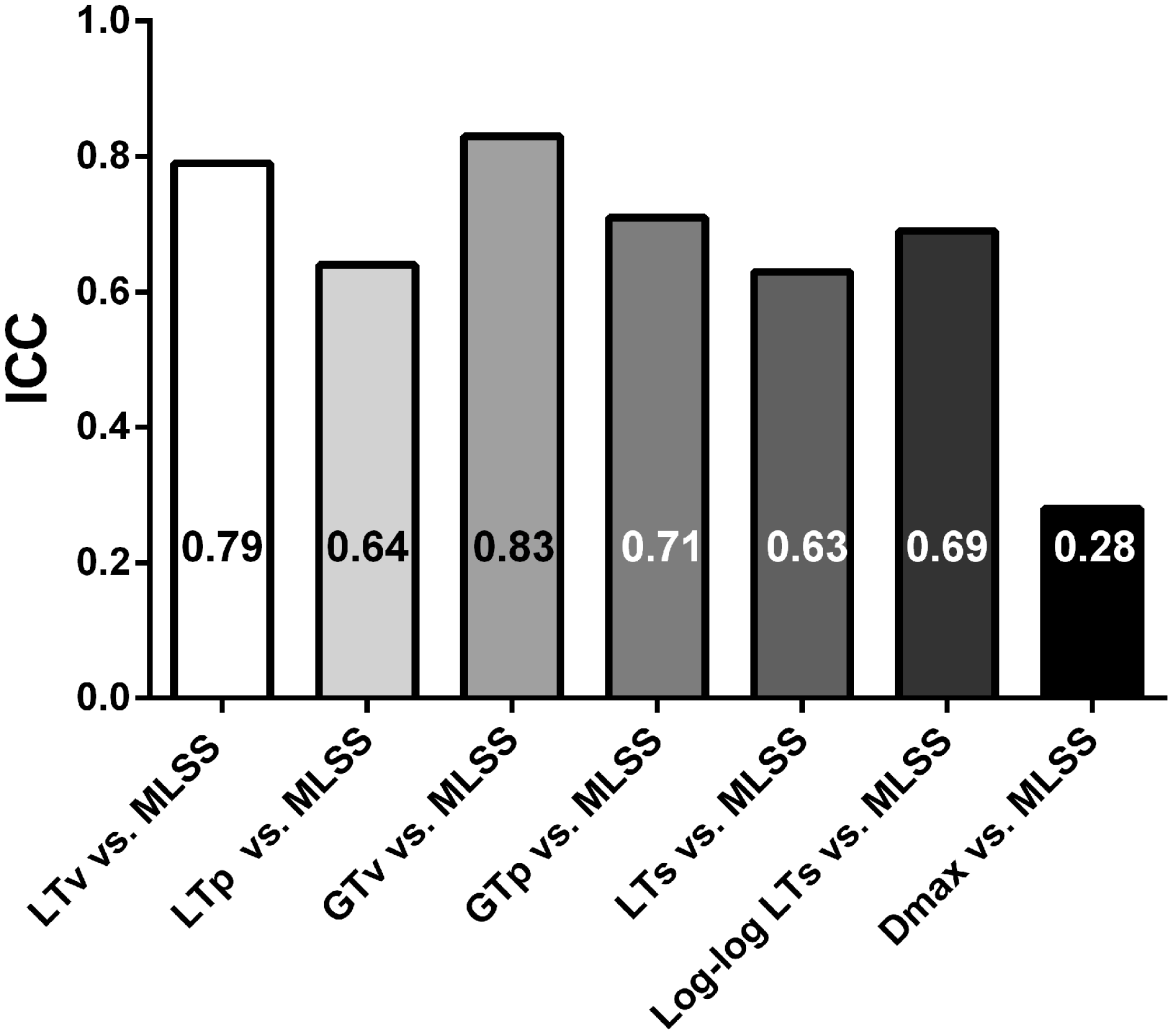
**Intraclass correlation coefficients (ICC) of the lactate threshold (LT) and glycemic threshold (GT) velocities vs. the maximal lactate steady state (MLSS) velocity**. All correlations were significant at *P* < 0.01.

Figure 4 shows Bland-Altman plots of the LTv, LTp, GTv, GTp, LTs, Log-log LTs, and Dmax velocities. Data from both groups (i.e., lean and obese) and both moments (i.e., pre and post) were clustered for each variable. Based on the low bias and relatively narrow limits of agreement, the LTv, GTv, LTs, and Log-log LTs velocities showed the best agreement with the MLSS velocity. On the other hand, the GTp and Dmax velocities showed the worst agreement with the MLSS velocity, since its bias was significantly different than zero (Figure 4H). Additionally, the intraclass correlation between the MLSS velocity and the GTv velocity was the greatest, followed, in descending order, by the intraclass correlation with LTv, GTp, Log-log LTs, LTp, LTs, and Dmax velocities (Figure 5). The intraclass correlation between the identification by assessors one and two together and identification by assessor three was 0.89 and 0.98, for LTv and GTv respectively.

**Figure 6:**
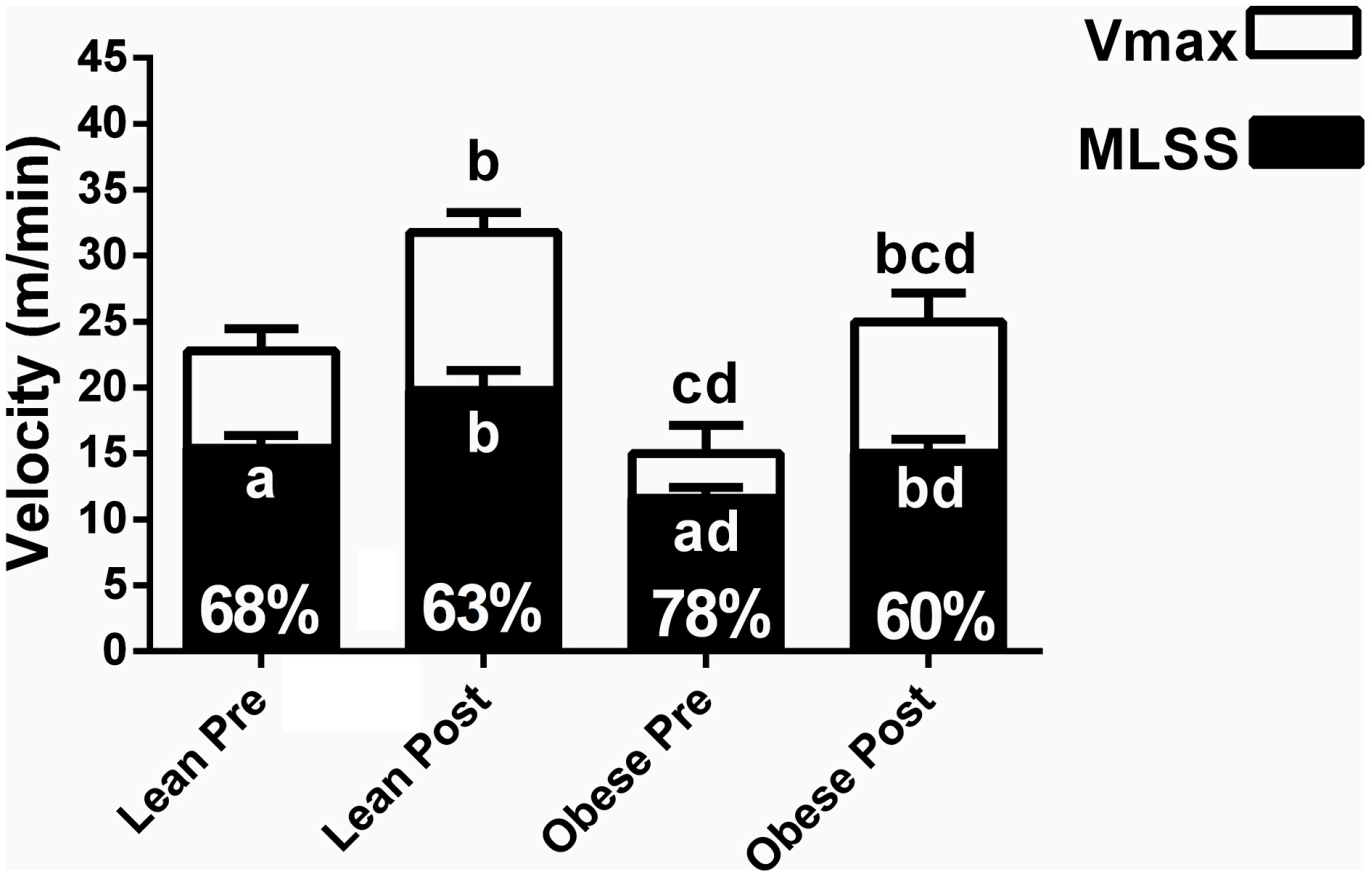
**Maximal velocity (Vmax) and velocity of the maximal lactate steady state (MLSS) for lean and obese rats at pre- and post-training**. The numbers inside the bars show the percentage that the MLSS velocity occurred in comparison with the Vmax. Letters inside the bars indicate *P* values for the MLSS. Letters above the bars indicate *P* values for the Vmax. ^*a*^*P* < 0.05 MLSS (%) vs. 60% within each group; ^*b*^*P* < 0.05 vs. pre within group; ^*c*^*P* < 0.05 vs. pre between groups; ^*d*^*P* < 0.05 vs. post between groups. ANOVA main effects: Vmax - group *P* < 0.0001, time *P* < 0.0001, interaction *P* = 0.03; MLSS - group *P* < 0.0001, time *P* < 0.0001, interaction *P* = 0.14).

**Figure 7:**
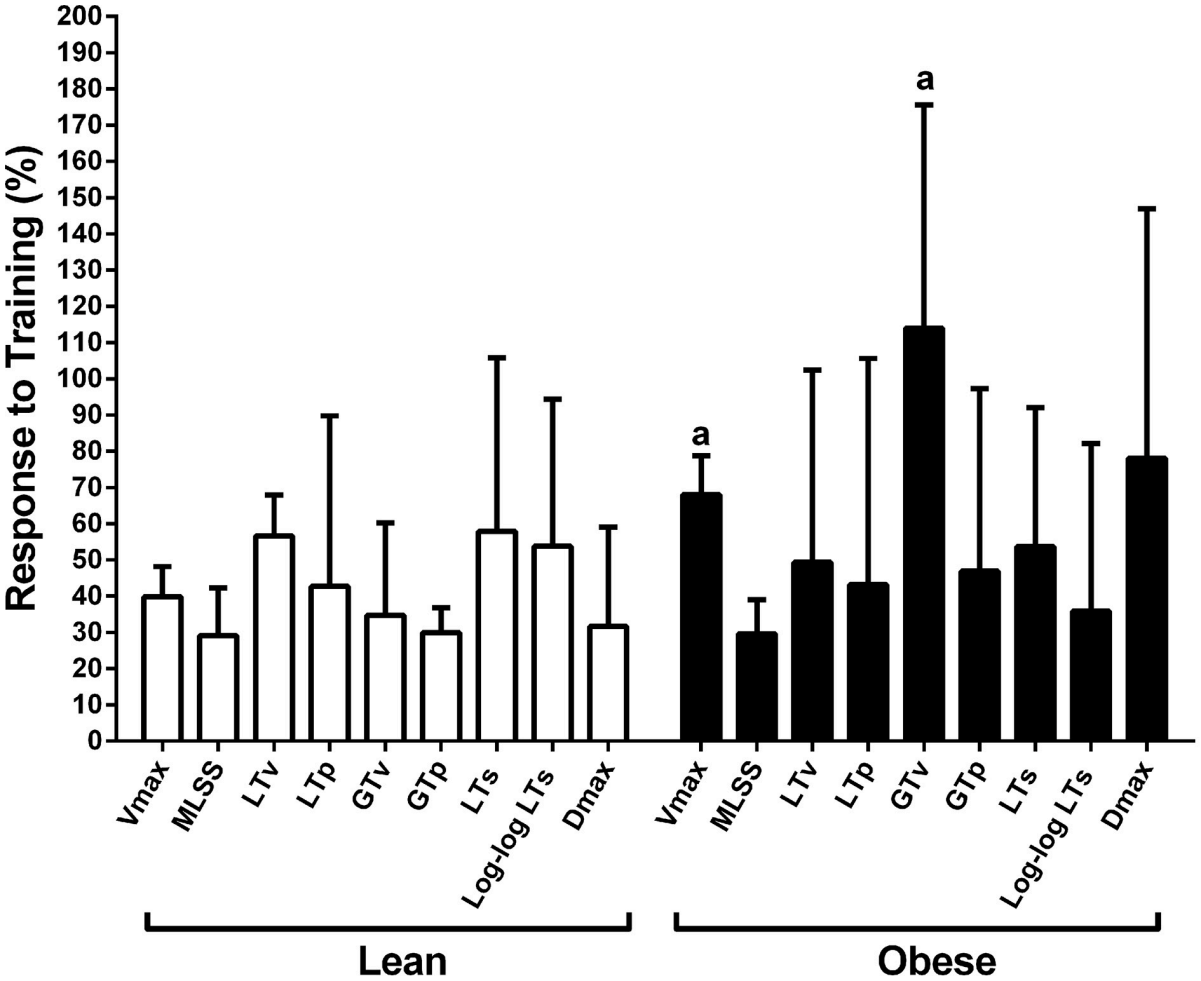
**Response to aerobic training calculated as (post–pre)/pre × 100**. ^*a*^*P* < 0.05 vs. lean.

The MLSS velocity corresponded to 68 ± 5%, 63 ± 7%, 78 ± 9%, and 60 ± 6% of the Vmax for lean pre-, lean post-, obese pre- and obese post-training, respectively (Figure 6). The MLSS velocity of lean and obese groups was significantly higher than 60% of the Vmax at pre-training. The MLSS velocity and Vmax were lower in the obese group at pre-training (Figure 6; *P* < 0.05 vs. pre), increased in both groups at post-training (*P* < 0.05 vs. pre), but were still lower in the obese group at post-training (*P* < 0.05 vs. lean). Training-induced increase (i.e., response to training) in MLSS, LT and GT velocities was similar between groups (*P* > 0.05), whereas increase in Vmax was greater in the obese group (*P* < 0.05 vs. lean; Figure 7).

## Discussion

The present study identified the MLSS on constant load exercise tests and the LT, GT, and Vmax on an incremental load exercise test before and after 8 weeks of treadmill training in Zucker rats. The main findings that arouse from this approach were: (1) in general, the LT and GT velocities were similar to the MLSS velocity at pre- and post-training; (2) the MLSS velocity was significantly higher than 60% of the Vmax at pre-training, but approached 60% of the Vmax at post-training, particularly in the obese rats; (3) aerobic training-induced increase in MLSS, LT, and GT velocities was similar between obese and lean rats, whereas the Vmax increase was larger in the obese rats.

Studies in Wistar rats (Cunha et al., 2009) and ob/ob mice (Almeida et al., 2011) found that the LT and MLSS occur at similar exercise intensity. Our study adds that the LT and GT exercise intensity also coincide with the MLSS exercise intensity in Zucker rats. The coincidence of the LT, GT and MLSS exercise intensity in rodents is quite different from findings in healthy humans and humans with grade 1 obesity (Simões et al., 2013). Thus, translation of data from rodents to humans should take into account that the LT and GT exercise intensity in rodents, particularly in Zucker rats, represents the upper limit of the heavy intensity domain.

A possible explanation for the coincidence between the LT and MLSS velocities in our study is the increased basal level of [Lac], which was close to the 4 mM break point, and thus above the level where the first inflection may occur in lean humans (Billat et al., 2003; Faude et al., 2009). The large increase in the adipose tissue of obese rats, disproportional to the increase in capillarity, may lead to hypoxia in the adipose tissue (DiGirolamo et al., 1992; Hodson, 2014; Trayhurn, 2014), increasing anaerobic glycolysis and lactate production (DiGirolamo et al., 1992; Hodson, 2014; Trayhurn, 2014). In addition, the high level of [Lac] and [Gluc] at rest in these rats can be attributed to high sympathetic activity (De Angelis et al., 2009). Noteworthy, it is improbable that the high [Lac] and [Glu] at rest was attributed to stress of blood sampling, because the [Lac] and [Glu] remained at resting levels during the early stages of the incremental test. If the [Lac] and/or [Glu] were artificially high at rest, it would probably reduce during low intensity exercise (Tegtbur et al., 2001; Voltarelli et al., 2002).

Among many different methods to identify the LT and GT, our results specifically showed that the LTv, Log-log LTs and LTs were the best methods to identify the LT velocity, and that the GTv was the best to identify the GT velocity. This is because these methods coincided with the MLSS velocity in most analyses and presented the highest intraclass correlation coefficients with the MLSS velocity, as well as the best agreement in Bland-Altman plots. The advantage of the LTv and the GTv is that they are practical to be done through visual inspection. Nevertheless, both depend on subjectiveness and experience of an evaluator to identify the inflection on the [Lac] or [Glu] curve. Based on our data, in addition to visual inspection, the Log-log LTs and LTs could be used to objectively confirm the identification of the LTv velocity. On the other hand, our results showed that the LTp, GTp, and Dmax velocities did not present satisfactory agreement with the MLSS velocity, and thus should not be used in Zucker rats. It seems that the reason why these methods did not coincide with the MLSS is that they take into account data obtained close to the maximal exercise capacity. At this point, data dispersion increases, as shown in Figure 2, which may shift thresholds identification.

In our study, we found that the MLSS velocity of the obese group was lower than the lean group. This result is similar to a previous study, which compared obese Zucker rats with Wistar rats (Almeida et al., 2013). Moreover, we found that the MLSS velocity of the obese group corresponded to higher percentage of the Vmax than the lean group, and that the MLSS velocity was significantly higher than 60% of the Vmax in both groups at pre-training. This illustrates that the same percentage of the Vmax can represent different exercise domains for obese vs. lean Zucker rats, and for Zucker rats vs. other rat strains. This may be attributed to higher body weight and lower relative muscle mass in Zucker rats, particularly in the obese Zucker (Leonard et al., 2005).

Almeida et al. (2014) showed that 4 weeks of treadmill training at the MLSS velocity increased the MLSS velocity by 20% in obese Zucker rats. In our study, lean and obese groups showed similar improvement in the MLSS velocity after 8 weeks of treadmill training (lean 28.5% and obese 29.3%). Therefore, it seems that most of the improvement that was observed in our study probably occurred in the first 4 weeks of training, than the last 4 weeks. This may have occurred in our study because duration and intensity of exercise sessions were maintained from the third to the 8th week. We did not adjust training load because there was no report in the literature of an incremental test to determine intensity domains in Zucker rats. Our data, in turn, bring support for the use of incremental treadmill test in Zucker rats, which will probably be useful to adjust the training load in future studies. Almeida et al. (2014) also suggested that obese Zucker rats showed attenuated increase in aerobic capacity in comparison with Wistar Kyoto rats. However, these rats have different genetic background, which affect data interpretation. Our study compared obese Zucker rats with lean Zucker rats, and found that improvement in submaximal aerobic capacity was similar between groups. This shows that training adaptation is not blunted by severe obesity *per se* in Zucker rats, which is a relevant finding that should be confirmed by further studies in humans with morbid obesity.

The MLSS velocity occurred at a high percentage of the Vmax at pre-training in the obese group (i.e., 78% of the Vmax). Hypothetically, this may be attributed to characteristics such as lower muscle glycogen content (Leonard et al., 2005), microvascular dysfunction (Butcher et al., 2014), lower perfusion in skeletal muscles (Butcher et al., 2014), and lower muscle mass (Nilsson et al., 2013; inherent characteristics in the obese Zucker rat) that may interfere in the ability of the animal to support progressive intensities above the MLSS. Data indicate that humans with morbid obesity show anaerobic threshold (determined by the V-slope method) between 72.9 and 78.5% of the maximal intensity achieved on an incremental test (Li et al., 2001), which is similar to the results from our study. On the other hand, humans with obesity grade 1 show anaerobic threshold [identified as the intensity at which lactate increased 1 mM above baseline on an incremental test] at approximately 35% of the maximal exercise capacity (Bircher and Knechtle, 2004). This demonstrates that obese Zucker rats have aerobic capacity particularly similar to that found in humans with morbid obesity, favoring their use to design translational studies.

Aerobic training increased both the MLSS velocity and the Vmax in obese rats, but the Vmax increase was larger. Then, at post-training, the MLSS velocity occurred at a percentage of the Vmax that was similar to the lean group. This indicates that 8 weeks of exercise training corrected the disparity between the MLSS velocity and the Vmax in obese Zucker rats. Perhaps the effect of aerobic training on muscle perfusion and muscle mass may have been greater in obese Zucker rats, which may have increased their ability to support progressive intensities above the MLSS. Noteworthy, strong evidence points an inverse association between aerobic capacity and risk of morbidity and mortality in several populations (Kokkinos and Myers, 2010), including patients with morbid obesity (McCullough et al., 2006). Therefore, the maximal aerobic capacity improvement in the obese group in our study may have relevant clinical implications, such as reduced morbidity and mortality, which should be further investigated.

One of the limitations of the present study was the absence of an untrained control group. However, a previous study showed that body weight of sedentary obese Zucker rats increased after 4 weeks of follow-up (3.3% per week), whereas the MLSS velocity did not change (Almeida et al., 2014). Therefore, it indicates that the decrease in body weight gain (0.6% per week) and improvement in aerobic capacity in our Zucker rats were attributed to the exercise training *per se*, but not to the follow-up period. The present study assessed only male rats, and it is known that male Zucker rats are more likely to develop metabolic abnormalities (e.g., higher insulin resistance and lipogenesis) than female counterparts (Macotela et al., 2009). At last, gas exchange was not measured, which could have enriched the study's interpretation.

In conclusion, MLSS, GT, and LT occurred at similar exercise intensity in obese Zucker rats at pre- and post-training, which seems to be a common characteristic of rodents, and thus should be taken into account to translate rodent data to humans. This exercise intensity was closer to the Vmax at pre-training than the one commonly reported for other rat strains. Besides, severe obesity did not attenuate aerobic training-induced increase in submaximal and maximal aerobic capacity.

## Author contributions

Study design: TR, HS, MR, BS. Conceived and carried out experiments: TR, HS, MR, MM, BD, RA, BS. Biochemistry analisys: TR, BD. Statistical analysis: TR, BS. Writing the paper: TR, HS, MR, MM, BD, MA, BS. Study advisor: BS.

### Conflict of interest statement

The authors declare that the research was conducted in the absence of any commercial or financial relationships that could be construed as a potential conflict of interest.

## Abbreviations

ANOVA: analysis of variance
[Gluc]: glucose concentration
[Lac]: lactate concentration
Dmax: maximal deviation
GT: glycemic threshold
GTp: polynomial adjustment of glycemic threshold
ICC: intraclass correlation coefficient
Log-log LTs: logarithm adjustment of lactate threshold
LT: lactate threshold
LTp: polynomial adjustment of lactate threshold
LTs: software adjustment of lactate threshold
MLSS: maximal lactate steady state
NaF: sodium fluoride
QGluc: glucose quotient
QLac: lactate quotient
Vmax: maximal velocity.
